# Differential DNA Methylation in Prostate Tumors from Puerto Rican Men

**DOI:** 10.3390/ijms22020733

**Published:** 2021-01-13

**Authors:** Gilberto Ruiz-Deya, Jaime Matta, Jarline Encarnación-Medina, Carmen Ortiz-Sanchéz, Julie Dutil, Ryan Putney, Anders Berglund, Jasreman Dhillon, Youngchul Kim, Jong Y. Park

**Affiliations:** 1 Department of Basic Sciences, Ponce Research Institute, Ponce Health Sciences University-School of Medicine, Ponce 00716-2347, Puerto Rico; gruiz@psm.edu (G.R.-D.); jencarnacion@psm.edu (J.E.-M.); carmenortiz@psm.edu (C.O.-S.); jdutil@psm.edu (J.D.); 2 Department of Biostatistics and Bioinformatics, H. Lee Moffitt Cancer Center, Tampa, FL 33612, USA; ryan.putney@moffitt.org (R.P.); anders.berglund@moffitt.org (A.B.); youngchul.kim@moffitt.org (Y.K.); 3 Department of Pathology, H. Lee Moffitt Cancer Center, Tampa, FL 33612, USA; Jasreman.Dhillon@moffitt.org; 4 Department of Cancer Epidemiology, H. Lee Moffitt Cancer Center, Tampa, FL 33612, USA; jong.park@moffitt.org

**Keywords:** prostate cancer, DNA methylation, aggressive prostate cancer, indolent prostate cancer, Gleason score, Hispanic/Latino, DNA repair, ancestry

## Abstract

In 2020, approximately 191,930 new prostate cancer (PCa) cases are estimated in the United States (US). Hispanic/Latinos (H/L) are the second largest racial/ethnic group in the US. This study aims to assess methylation patterns between aggressive and indolent PCa including DNA repair genes along with ancestry proportions. Prostate tumors classified as aggressive (*n* = 11) and indolent (*n* = 13) on the basis of the Gleason score were collected. Tumor and adjacent normal tissue were annotated on H&E (Haemotoxylin and Eosin) slides and extracted by macro-dissection. Methylation patterns were assessed using the Illumina 850K DNA methylation platform. Raw data were processed using the Bioconductor package. Global ancestry proportions were estimated using ADMIXTURE (k = 3). One hundred eight genes including *AOX1* were differentially methylated in tumor samples. Regarding the PCa aggressiveness, six hypermethylated genes (*RREB1, FAM71F2, JMJD1C, COL5A3, RAE1,* and *GABRQ*) and 11 hypomethylated genes (*COL9A2, FAM179A, SLC17A2, PDE10A, PLEKHS1, TNNI2, OR51A4, RNF169, SPNS2, ADAMTSL5,* and *CYP4F12*) were identified. Two significant differentially methylated DNA repair genes, *JMJD1C* and *RNF169*, were found. Ancestry proportion results for African, European, and Indigenous American were 24.1%, 64.2%, and 11.7%, respectively. The identification of DNA methylation patterns related to PCa in H/L men along with specific patterns related to aggressiveness and DNA repair constitutes a pivotal effort for the understanding of PCa in this population.

## 1. Introduction

Since 1984, prostate cancer (PCa) has been the most commonly diagnosed cancer in the United States (US), currently accounting for 19% of all cancers in men. Approximately 12.1% of men will be diagnosed with PCa in their lifetime [1]. In 2020, approximately 191,930 new PCa cases and 33,330 deaths are estimated in the US [2]. Hispanic/Latinos (H/L) are the second largest racial/ethnic group in the US after non-Hispanic Whites (NHWs). Chinea et al. (2017) reported that H/L subgroups have different prostate cancer-specific mortality (PCSM) rates when compared to NHWs and non-Hispanic Blacks (NHBs) using data from 2000–2013 that included 486,865 men. PCa incidence and mortality rates in H/L men were similar to NHWs [3]. However, these data may be overgeneralized because all H/L subgroups are aggregated into one broad group. Data from the Puerto Rico (PR) Cancer Registry show that PCa is the leading cancer type, both in terms of incidence (39.9% of all cancer cases) and in terms of mortality (18.3% of all cancer deaths) [4]. A recent study reported that Puerto Rican H/L (PR H/L) men have a higher rate of survival and treatment outcomes than NHWs [3]. However, PR H/L men had significantly higher PCSM than NHBs and the highest mortality among Hispanic subgroups [3].

PCa diagnosis is mainly based on the evaluation of biopsied tissue by a pathologist that produces a Gleason score of disease severity. This is associated with an average error of 25–30% in the case of under-detection and an average error of 1.3–7.1% in the case of over-detection [5,6]. The accuracy of a Gleason score is estimated to be 61% [7,8]. Although PCa is normally characterized by a slow progression, about 20–30% of cases are associated with an aggressive phenotype that could lead to metastasis and death. A key molecular feature of this aggressive phenotype is the dysregulation of tumor suppressor genes and DNA repair genes. Defects in DNA repair pathways in PCa can be effectively targeted using PARP1 inhibitors. Prostate tumors with deficiencies in *BRCA1* or *BRCA2* DNA repair genes are highly sensitive to these drugs [9]. Although alterations of these genes in prostate tumors have been studied [10,11], little is known about the epigenetic regulation of DNA repair genes in PCa. No published data are currently available regarding DNA methylation in PR H/L PCa patients.

Epigenetic changes and modifications represent critical components of initiation and progression of carcinogenesis [12]. Abnormal epigenetic programs, including DNA methylation may inactivate large groups of genes. Hundreds of epigenetically silenced genes may exist in tumor tissues [13]. DNA methylation has been extensively studied, and hypermethylation has been linked with gene silencing of tumor suppressor genes in PCa and with adverse clinical outcomes [12,14,15,16]. DNA methylation is an epigenetic process that affects transcriptional regulation of genes. DNA methylation occurs when a methyl branch is added to position 5 of cytosine, and normally 3–4% of all cytosines are methylated [13]. Methylation only occurs at cytosine nucleotides located 5′ to guanine nucleotides forming CpGs or their clusters, termed CpG islands. DNA methylation is part of a cluster of molecular processes that initiate tumorigenesis and drive its early evolution by altering other molecular processes.

We previously reported that numerous studies have investigated DNA methylation in PCa [17,18]. These studies reported a number of differentially methylated genes in PCa compared to adjacent normal tissues. Most of these studies used a candidate gene approach advantage to increase statistical efficiency of the study association analysis while narrowing the possibilities of assessing methylation effects related to multiple genes. Several studies used an epigenome-wide methylation microarray to cover a wide range of genes. All studies identified a large number of differentially methylated CpGs, but these findings have not been confirmed in independent validation datasets [19,20,21]. Since DNA methylation changes may drive racial and ethnic disparities in PCa [22], there is a need to study these patterns from different small cohorts to identify potential candidates to further conduct representative validation studies.

This study aimed to investigate epigenetic differences in terms of DNA methylation between tumor and adjacent normal prostate tissues. Moreover, we aimed to assess differences in DNA methylation patterns in aggressive (high risk) and indolent (low risk) PCa in order to identify specific biomarkers for the aggressive phenotype in PR H/L men. A secondary aim was to characterize the ancestry component of men in this study group. PR has an admixed population consisting of three main components: European, African, and Indigenous American.

## 2. Results

### 2.1. Clinicopathological Characteristics of Study Group

As shown in Table 1, PR H/L men in the aggressive (high-risk) group had a mean age of 65.5 and were significantly older than those in the indolent (low-risk) group, with a mean age of 59.4 years. As expected, because of the selection criteria used, the high-risk group showed significant differences in Gleason scores as compared with the low-risk group. No differences between the two groups were evident in terms of prostate-specific antigen (PSA) levels, ethnicity, vital characteristics, biochemical recurrence, tumor stage, surgical margins, and family history of prostate cancer (*p* > 0.05).

### 2.2. Differences in Methylation Levels between Tumors and Adjacent Normal Tissue

**Figure 1:**
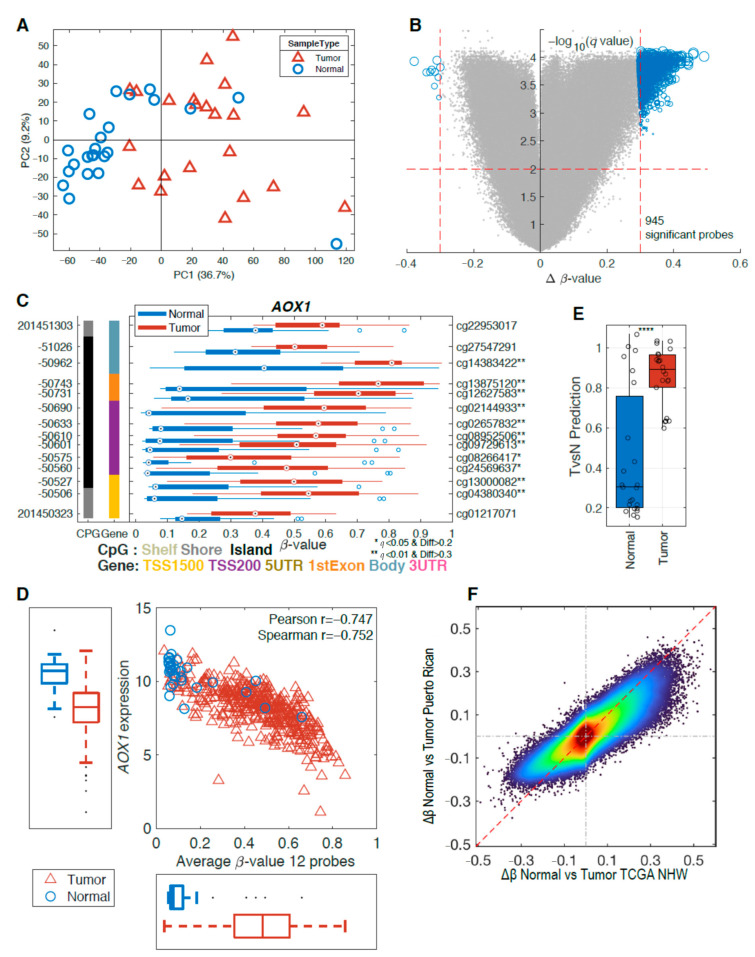
Methylation differences in tumors vs. normal tissue. (**A**) Principal component analysis (PCA) of 45 samples in our study shows a separation between tumor (red triangles, *n* = 22) and adjacent normal tissue (blue circles, *n* = 23). (**B**) The volcano plot above indicates changes in the methylation pattern between normal and tumor tissues with hypermethylated regions in tumors. (**C**) Gene structure methylation (GSM) plot for a representative gene, *AOX1*. The methylation level (*x*-axis) for each probe set is represented by a boxplot for each group with tumors in red and normal in blue. The genomic and the probe identifier (ID) are shown on the left and right *y*-axis, respectively. The far-left column (black) indicates the presence of a CpG island and the next column (colored) shows the CpG probe location in the gene. Multiple probes showed significant differential methylation level. (**D**) Average methylation level for 12 probes vs. RNA-seq gene expression levels show a negative correlation using The Cancer Genome Atlas Prostate Adenocarcinoma (TCGA-PRAD) non-Hispanic white (NHW) samples. (**E**) Partial least squares (PLS) model derived using TCGA NHW samples can clearly separate the samples from the Puerto Rico (PR) cohort. (**F**) Density scatter plot of Δβ-values for overlapping probes for the TCGA Illumina 450K probes vs. PR cohort (EPIC) probes. **** *p* < 0.0001.

An initial quality control of our samples considering the missing values, β-value distributions, and principal component analysis (PCA) indicated that three samples (two tumor and one normal) did not meet our quality control and were excluded from further analysis. The unsupervised PCA model of the remaining samples showed a clear distinction between tumor and normal tissues in the first principal component (PC1), explaining 36.7% of the variation (Figure 1A). This indicates that there are considerable methylation changes between normal and tumor tissue. To further explore the methylation differences between normal and tumor tissue, we performed a two-group comparison. The false discovery rate (FDR)-corrected *p*-values (*q* < 0.01) and the mean difference (Δβ-value > 0.3) between the two groups are presented in a volcano plot (Figure 1B). This analysis resulted in 945 significant probes, with a majority of the probes showing hypermethylation in tumor samples (Figure 1B). To identify differently methylated genes, we further required two consecutive probes within a gene to be significant and this resulted in 108 differently methylated genes (Supplementary Materials, Table S1). One of identified genes, *AOX1*, which was previously suggested as a methylation biomarker for PCa [21,23], showed significant hypermethylation for multiple probes in tumor tissue (Figure 1C). Using data from The Cancer Genome Atlas (TCGA), we show that this aberrant promoter methylation led to downregulation of *AOX1* in tumor tissues (Figure 1D).

To further investigate our data, we integrated the methylation results on a partial least squares (PLS) model for tumor vs. normal using only non-Hispanic White (NHW) samples from The Cancer Genome Atlas Prostate Adenocarcinoma (TCGA-PRAD) dataset. Applying this PLS model to our data, we can observe that samples of Puerto Rican origin show similar methylation changes when comparing tumor and normal tissues for the two datasets (Figure 1E). This is further established (Figure 1F) when comparing the Δβ-value between tumors and normal for TCGA NHWs and this PR cohort, which shows a clear linear tendency. Of the 945 significant probes found in the PR cohort, 799 are available on the 450K chip used by the TCGA and 585 (73%) of these were also significant in the TCGA NHW cohort.

### 2.3. Differentially Methylated Genes Associated with Aggressive Prostate Cancer

**Figure 2:**
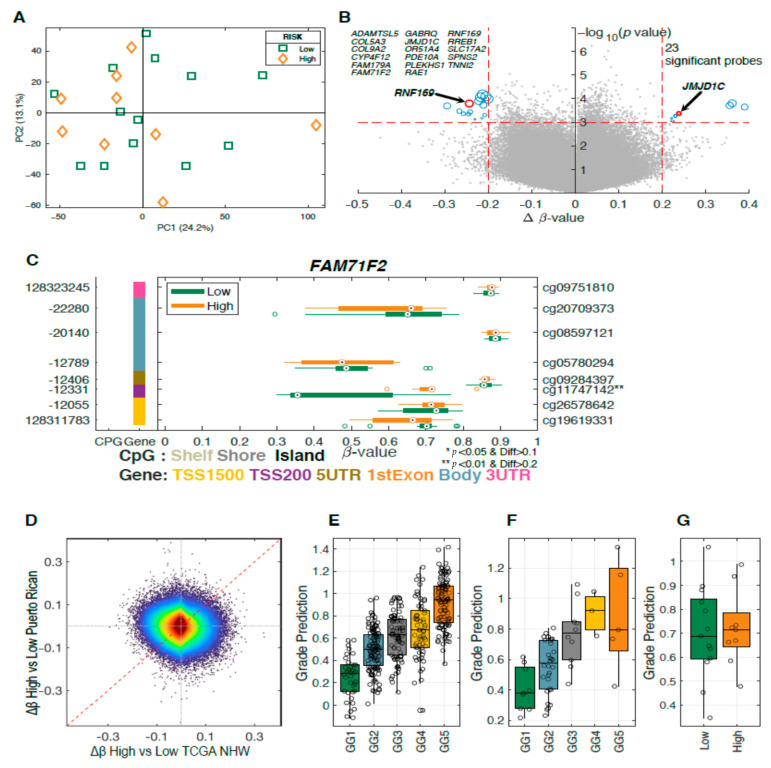
Methylation differences in high-grade vs. low-grade prostate tumors. (**A**) PCA of 22 samples in our study shows no separation between low-risk (green rectangles, *n* = 13) and high-risk (orange diamonds, *n* = 9) groups. (**B**) Volcano plots highlighting 23 differentially methylated sites between high-risk and low-risk prostate tumors with a −log_10_ *p*-value on the *y*-axis and Δβ-value on the *x*-axis. (**C**) GSM plot for *FAM71F2* demonstrating lower level of methylation for the low-risk group compared to the high-risk group for cg11747142. (**D**) Density scatter plot comparing the Δβ-value for GG1 vs. GG5 for the TCGA PRAD NHW samples compared to the Δβ-value for the high-risk vs. low risk Puerto Rican samples. Prediction results for a PLS model derived using (**E)** TCGA PRAD NHW GG1 vs. GG5 applied to the TCGA NHW samples, (**F**) TCGA non-Hispanic Black (NHB) samples, and (**G**) Puerto Rican samples.

To further investigate the differentially methylated genes in prostate tumor samples, we calculated a PCA model including 22 tumor samples identified as aggressive (high-risk) and indolent (low-risk). No clusters were found on the sample score plot between aggressive and indolent cases (Figure 2A). No significant results were obtained when comparing between the indolent and aggressive groups using FDR (0.84). However, 23 probes were identified as potential candidates using a *p* < 0.001 and a Δβ-value of 0.2 as cutoff (Figure 2B and Table 2).

### 2.4. Methylation Analysis of DNA Repair Genes Associated with Aggressive Prostate Cancer

The volcano plot shows the significant differentially methylated CpG sites between aggressive and indolent prostate tumors found in 17 genes (Figure 2B). Six of these genes were hypermethylated (*RREB1, FAM71F2, JMJD1C, COL5A3, RAE1, and GABRQ*), and 11 were hypomethylated (*COL9A2, FAM179A, SLC17A2, PDE10A, PLEKHS1, TNNI2, OR51A4, RNF169, SPNS2, ADAMTSL5,* and *CYP4F12*). Table 2 presents the location of CpG sites, mean methylation levels in high- and low-risk groups and the *p*-values for these differentially methylated genes. One of the identified probes mapped to the gene named *FAM71F2* (family with sequence similarity 71 member F2), which was previously suggested as a biomarker for metastasis of testicular cancer [24]. Probe cg11747142 was found to be significantly hypermethylated in tumor tissues (Figure 2C).

To compare our results with previous studies, we also included results from the NHW samples in the TCGA PRAD dataset (Figure 2E). The Δβ-values for comparing Grade Group 1 (GG1) vs. Grade Group 5 (GG5) are compared to our Δβ-value in Figure 2D. It is clear that there is no consistency in differently methylated probes between the two datasets, opposite to what was seen in Figure 1F. To further compare the two datasets, we derived a PLS model for the NHW samples using the GG1 vs. GG5. This model was applied to all the Grade Groups for the TCGA NHW samples (Figure 2E). For the sample used in the PLS model, GG1 and GG5 are clearly separated but there is also a clear trend difference among the GG2, GG3, and GG4 groups which were not part of the PLS model training set. To further validate our PLS model, we also applied it to the TCGA PRAD NHB samples (Figure 2F), and these predictions showed a similar trend as for the NHW samples. This would indicate that the NHW-derived PLS models also work on NHB samples. The PLS model was also applied to the PR cohort. However, it could not separate low-risk from high-risk samples (Figure 2G).

**Figure 3:**
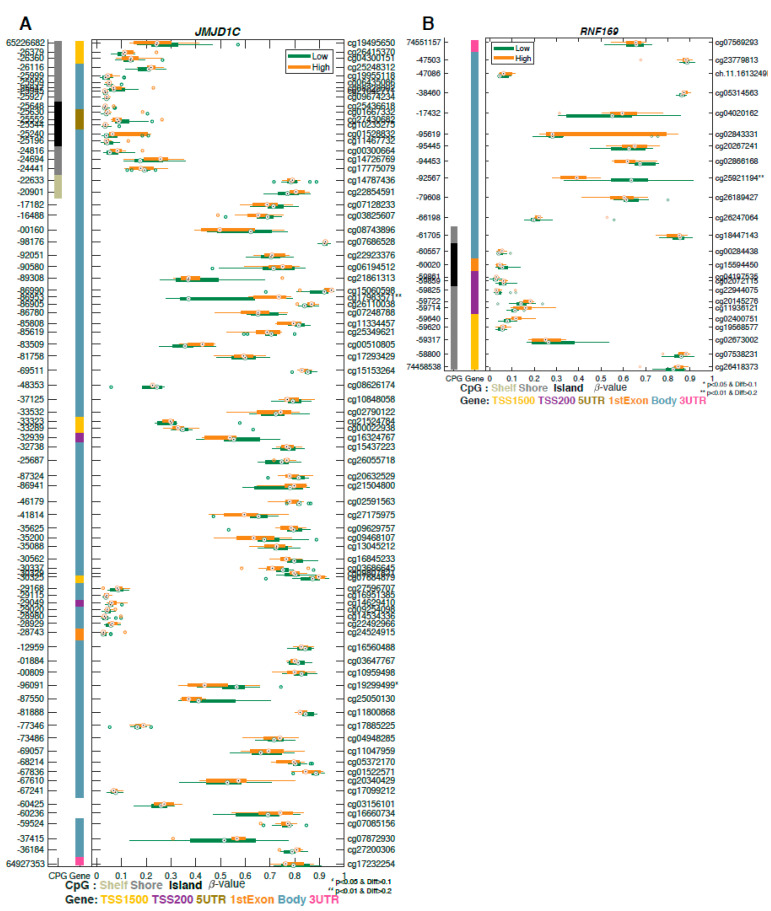
Two differentially methylated DNA repair genes associated with aggressiveness were found to contain probes with significant changes in methylation. GSM plots for (**A**) *JMJDC1* and (**B**) *RNF169* are shown.

We analyzed differential methylation of 179 candidate DNA repair genes between tumor and normal tissues and between aggressive and indolent cases. These genes were predominantly distributed in five DNA repair pathways including nucleotide excision repair, base excision repair, mismatch repair, and homologous and nonhomologous repair. We found two significant differentially methylated DNA repair genes: *JMJD1C* and *RNF169* (Figure 2B). *JMJD1C* (Figure 3A) codifies for a histone demethylase protein which plays a role in regulation of MDC1 protein expression [9]. *RNF169* (Figure 3B) acts as a negative regulator of double-strand break (DSB) repair following DNA damage [25].

### 2.5. Ancestry Analysis

**Figure 4:**
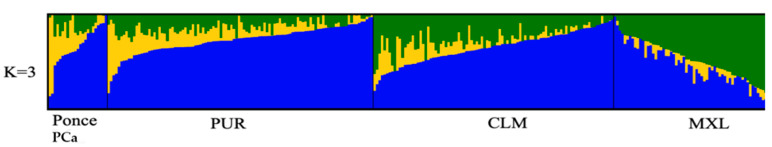
Visualization of the ancestry proportions for each individual in 23 PR H/L men with PCa (Ponce PCa) compared to 1000 Genomes Admixed Americans populations. Assuming three ancestral populations (k = 3), each column represents one individual, and each color corresponds to the contribution of each ancestral population to the genome of a given individual (blue = European, yellow = African, and green = Indigenous American). PUR Puerto Ricans from Puerto Rico, MXL Mexican Ancestry from Los Angeles, CLM Colombians from Medellin, Colombia, and PEL Peruvians from Lima, Peru from the 1,000 Genomes project.

For each study participant, we determined the proportion of African, European, and Indigenous American ancestry. As shown in Table 3, the contribution of African ancestry ranged between 2.7% and 85.8%, averaging 24.1% (standard deviation, SD 22.6%). For the European ancestry component, values ranged between 9.4% and 97.1%, averaging 64.2% (SD 21.1%). For the Indigenous American, values ranged between 0% and 25.16%, averaging 11.7% (SD 9.0%). It is noteworthy that, within this population, there are large variations in the contribution of European versus African ancestry, while the Indigenous American ancestry remains relatively homogenous (Table 3, Figure 4). This contrasts with what has been observed in H/L of Mexican and Peruvian origin for which there is a stronger influence of Indigenous American ancestry.

## 3. Discussion

This pilot study represents the first effort to study epigenetic regulation in PR H/L men with PCa. Several potential DNA methylation biomarkers for aggressive and indolent prostate tumors were identified. Our results integrate data from TCGA NHW and NHB which provides a perspective regarding methylation patterns in PR H/L men. Moreover, two differentially methylated protein-coding genes associated with DNA repair were identified, shedding light on the differences between aggressive and indolent PR H/L men with PCa. The outcomes of this study could lead to the development of better methods for clinicians to identify PR H/L men with PCa with low and high risk.

Our study is not the first to identify differences in methylation patterns between tumor and adjacent normal tissue in PCa. Using epigenome-wide 450K DNA methylation data derived from 469 PCa tumors and 50 normal prostatic tissue samples, Nikas and Nikas (2019) were able to develop a mathematical model that classified prostate tumor tissue versus normal tissue with a high sensitivity (95.3%) and specificity (94.0%) [8]. Since this group only compared tumor with adjacent normal tissues, their results cannot reflect differences within prostate tumors (aggressive and indolent). Our study is an effort to combine adjacent normal and tumor tissue to explore other differences that might be related to biological processes. Xu et al. (2019) investigated 553 PCa tumor samples in the TCGA database associated with DNA methylation-driven genes between tumor and normal samples. Here, we show that there are common methylation patterns between PR H/L and NHWs [12].

One of identified genes, *AOX1*, showed significant hypermethylation for multiple probes in tumor tissue. The most promising methylation marker candidates identified by Strand et al. (2014) included *PITX2*, *C1orf114* (*CCDC181*), and the GABRE~miR-452~miR-224 locus, in addition to the three-gene signature *AOX1/C1orf114/HAPLN3* [23]. The function of *FAM71F2* is unknown although this gene is expressed in various tissues including testis, brain, and others. Gene expression analysis in testis cancer identified family with sequence similarity 71, member F2 (*FAM71F2*), which can discriminate the metastasis status with an excellent predictive significance [24].

Among 17 differentially methylated genes in aggressive cases, a few genes, such as *SLC17A2* and *OR51A4*, were not investigated previously for human cancers. However, most genes were investigated for a role in the methylation process, DNA repair, or differential expression in various tumors, including prostate cancer. 

Differential expression of *COL9A2* in prostate tumor was reported as compared in normal tissues. *COL9A2* was found as one of the hub genes in a protein–protein interaction network [26]. The zinc finger protein Ras-responsive element binding protein (RREB-1) interacts with androgen receptor (AR) as a partner and coregulator. The *RREB-1* gene binds to the prostate-specific antigen (PSA) promoter. Inhibition of *RREB-1* expression leads to increased PSA promoter activity and expression. Therefore, *RREB-1* acts as a repressor of AR [27,28]. *CYP4F12* is expressed differentially in colon [29] and liver tumor tissues [30,31]. Expression of *CYP4F12* is positively correlated with low clinical stages and is a prognostic biomarker for overall survival in liver cancer. These results suggest the potential predictive diagnostic and prognostic roles of *CYP4F12* gene expression in liver cancer [30].

Gene expression profiling analysis showed that *COL5A3* overexpression is related to breast cancer progression [32]. *ADAMTSL5* was proposed as a putative epigenetic marker for therapeutic resistance in acute lymphoblastic leukemia. Results from two methods, 27K microarray and methylation-specific polymerase chain reaction, showed hypermethylation of *ADAMTSL5* in chemo-resistant samples (93% vs. 38%; *p* = 0.0001) [33]. *RAE1* was identified as one of 23 genes involved in the transformation from androgen-dependent PCa to castrate-resistant PCa. These 23 genes play a role in important biological processes, such as cell signal transduction, cell adhesion, apoptosis, oncogenesis, cell proliferation, and cell differentiation [34].

Spinster homolog 2 (*SPNS2*) is a multi-transmembrane transporter, widely located in the cell membrane and organelle membranes. It transports sphingosine 1-phosphate (S1P) into the extracellular space and the circulatory system [35]. Sphingosine 1-phosphate (S1P) plays important roles in cell proliferation, differentiation, or survival mainly through its surface G-protein-coupled receptors S1P1–5. Bone represents the major site of metastasis for PCa cells, which rely on bone-derived factors to support their proliferation and resistance to therapeutics in PCa [36]. The *SPIN2* gene is known to be involved in angiogenesis, immune response, and metabolism. Furthermore, Spindlin Family Member2 (*SPIN2)* is associated with the transformation from inflammation to cancer and metastasis. A critical role of *SPIN2* was reported in the survival of lung cancer [37] and acute myeloid leukemia (AML) [38].

Troponin I2 (*TNNI2*) was identified as a candidate biomarker for prediction of poor outcomes in various cancers. Overexpression of *TNNI2* is associated with recurrence and metastasis in gastric cancer [39] and with poor survival in lung cancer [40]. *TNNI2* was hypomethylated in liver tumor [41]. Overexpression of Pleckstrin homology domain containing S1 (*PLEKHS1*) was associated with metastases, as well as shorter overall and disease-free survival in thyroid carcinoma. The messenger RNA (mRNA) expression of *PLEKHS1* was inversely correlated with methylation level. *PLEKHS1* plays a role in aggressive thyroid carcinoma and can be a biomarker for predicting poor outcomes [42].

The *FAM179A* was identified as a fusion partner to the anaplastic lymphoma kinase gene (ALK) in patients with non-small-cell lung cancer (NSCLC). Therefore, this *FAM179A–ALK* fusion may influence the treatment response to ALK inhibitors [43]. γ-Aminobutyric acid (GABA) A receptor subunit θ (*GABRQ*) is a well-known inhibitory neurotransmitter in the brain, suggested as a prognostic biomarker of clear cell renal cell carcinoma (ccRCC). Low *GABRQ* mRNA expression was significantly associated with a poor prognosis of ccRCC in two independent cohorts. *GABRQ* mRNA expression may be considered as a novel prognostic biomarker of ccRCC [44]. A previous study reported that *GABRQ* is involved in the risk and progression of liver cancer, which promotes the proliferation of cancer cells [45]. Phosphodiesterase10A (*PDE10A*) is expressed in prostate tissues. *PDE10A* was found frequently (≈19%) in novel somatic mutations. In silico analysis of this novel variant shows a possible alteration of *PCE10A* function [46]. A biological role for *PDE10A* has also been studied in neurodegenerative diseases and in colorectal cancer [47,48]. Therefore, PDE10A-specific inhibitors are investigated in preclinical studies [49].

Among 17 genes associated with aggressiveness, two genes are involved in the DNA repair processes. *JMJD1C*, a DNA repair factor, plays multiple important roles in prostate cancer progression. Downregulation of *JMJD1C* leads to an impact on the DNA repair pathway in the balance of homologous recombination (HR) and non-homologous end-joining (NHEJ) [9]. *JMJD1C* shows focal loss in PCa and may be associated with resistance to PARP inhibitors [9]. In addition, a PCa genome-wide association study (GWAS) identified a new locus, *JMJD1C* at 10q21, which was associated with serum testosterone levels (rs10822184: *p* = 1.12 × 10^−8^). This SNP in *JMJD1C* was estimated to account for 5.3% of the variance in serum testosterone and dihydrotestosterone levels. *RNF169* is involved in the DNA repair pathway via an interaction with dual-specificity tyrosine phosphorylation-regulated kinase 1A (DYRK1A), and it was reported to be involved in DNA double-strand break (DSB) repair [50,51]. *RNF169* is recognized as a substrate for *DYRK1A*. *RNF169* phosphorylation at S688 plays a major role in removing 53BP1 from the DNA damage foci [52]. Furthermore, *RNF169* interacts with *DYRK1A* and localizes to DNA damage foci by binding [53].

Differentially methylated probes (585) were found when comparing tumor vs. normal tissue in PR H/L samples and further confirmed using the TCGA data. Kyoto Encyclopedia of Genes and Genomes (KEGG) pathway enrichment analysis based on DNA methylation-driven genes obtained from the Gene Expression Omnibus (GEO) database did not report any DNA repair pathways as being enriched in PCa. However, recent genomic analysis revealed that germline or somatic inactivation mutations in *BRCA1* or *BRCA2*, or other genes involved in the homologous repair pathway collectively occur in as much as 20–25% of advanced PCa [54,55]. The identification of DNA methylation patterns related to PCa in PR H/L men along with specific patters related to aggressiveness and DNA repair constitutes a pivotal effort for the understanding of PCa disparities in this population. The study by Apprey et al. (2019) reported that genetic ancestry influences DNA methylation patterns [22]. This constitutes a unique effort to provide a broad overview regarding methylation and ancestry patterns in PR H/L men while opening new avenues for future studies.

## 4. Materials and Methods

### 4.1. Human Subjects, Sample Selection, and Specimen Acquisition

This study was approved by the Ponce Health Sciences University (PHSU) Institutional Review Board (Protocol no. 1909021277A001). All samples for this study were obtained from the Puerto Rico Biobank (PRBB), the only biorepository focused on the biobanking of Hispanic cancer patients from US. The PRBB is a core facility in Ponce Research Institute (PRI) and a key component of the U54 Partnership (PHSU/PRI-Moffitt Cancer Center) funded by the National Cancer Institute (NCI) Center to Reduce Cancer Health Disparities. Archived formalin-fixed paraffin-embedded (FFPE) blocks of 24 prostate tumor and 24 adjacent normal tissues were collected through the PRBB. We selected 11 aggressive and 13 indolent cases on the basis of Gleason scores.

### 4.2. DNA Methylation

#### 4.2.1. Illumina Methylation 850K Data Filtering and Genome-Wide Analysis Plan

The Illumina 850K DNA methylation platform (San Diego, CA) was used to measure DNA methylation patterns from FFPE isolated DNA according to the manufacturer’s instructions.

#### 4.2.2. DNA Extraction

DNA samples were extracted from the FFPE prostate tissues from the PRBB core at PHSU using a macro-dissection approach. Tumor areas were annotated on the H&E (Haemotoxylin and Eosin) slides by a pathologist (Dr. Jasreman Dhillon) and extracted by macro-dissection. To ensure the quality of the extracted nucleic acids, DNA extracted from the samples was evaluated for integrity with DNA integrity numbers (DINs).

#### 4.2.3. Quality Control (QC) and Normalization for Methylation Data

The ‘idat.files’ were read using the minfi (version 1.28.4) [56,57] Bioconductor package for R (version 3.5.2). Detection *p*-values were subsequently calculated using minfi’s implementation provided by the detectionP function. The function preprocessFunnorm was used to perform both background correction, using the normal-exponential out-of-band (NOOB) [58] method, and then functional normalization (FunNorm) [59], a between-array normalization method. The preprocessFunnorm function returned an object containing β-values which were calculated with an offset of 100 in the denominator, as suggested by Illumina [60]. The number of missing values, histogram of β-values, and principal component analysis (PCA) plots were used to visualize data quality and detect outlier samples and potential batch effects. β-Values with a corresponding detection *p*-value > 0.05 were set as missing values.

#### 4.2.4. Selection of Differentially Methylated Regions (DMRs)

Two group comparisons were performed using Student’s *t*-test controlling for multiple testing using FDR [61]. Furthermore, probes were considered significant if the difference in mean β-value between the two groups was larger than 0.3 [62]. To further remove potentially false positives, we used a region-based analysis. This is accomplished by simultaneously considering all the probes within a specific region (gene or CpG island) and defining a significant change when multiple probes within that region show the same significant change [63,64,65,66]. A single significant probe within a CpG island or gene body was not considered significant. Statistical analysis was performed using MATLAB (Natick, MA, USA).

#### 4.2.5. TCGA Data

Raw IDAT files were downloaded and normalized. RNA-seq data for PRAD was extracted from the PanCancer dataset (https://gdc.cancer.gov/about-data/publications/pancanatlas). Ancestry estimates were taken from the publication by Yuan et al. (2018) [67].

### 4.3. Ancestry Analysis

#### 4.3.1. Genotyping

A set of 106 single-nucleotide polymorphisms (SNPs) that can discriminate indigenous American, African, and European ancestry was used to estimate the proportion of genetic ancestry in 24 PR H/L PCa patients. The ancestry informative markers (AIMS) are widely spaced throughout the genome and have a well-balanced distribution across all 22 autosomal chromosomes, with an average distance between markers of 2.4 × 10^7^ bp. Genotyping of the ancestry informative markers was performed using a multiplex PCR coupled with single-base extension methodology with allele calls using a Sequenom analyzer at the University of Minnesota.

#### 4.3.2. Quality Control of Ancestry Data

Out of the 106 AIMS genotyped, five were excluded due to a genotyping rate <90% (*n* = 2, rs30125, rs10491097) and/or weak confidence in clustering (*n* = 3, rs993314, rs2585901, rs4076700). One individual with a call rate of <90% was excluded from further analysis.

#### 4.3.3. Ancestry Estimates

The global genomic ancestry proportion was estimated using Admixture under a supervised model and assuming three populations of origin (k = 3) using the -B (bootstrapping) flag to generate estimates of standard error. Reference populations for supervised analysis consisted of individuals of European ancestry (*n* = 42, Coriell’s North American Caucasian panel), African ancestry (37 non-admixed West Africans living in London, United Kingdom, and South Carolina), and Indigenous American (15 Mayans and 15 Nahuas).

## Figures and Tables

**Table 1:** Clinicopathological characteristics of Puerto Rican men with aggressive (high-risk) (*n* = 11) and indolent (low-risk) (*n* = 13) prostate cancer in the study group.

Risk	High	Low	*p*-Value ^1^
	*n* = 11	*n* = 13	
Age at diagnosis	65.5 ± 4.1	59.4 ± 10.5	0.006
PSA	8.3 ± 6.59	9.23 ± 9.33	0.350
Vital status			0.500
Deceased	1	0	
Alive	10	11	
Missing	0	2	
Gleason score			<0.0001
6	0	8	
7 (3 + 4)	0	5	
7 (4 + 3)	7	0	
8–9	4	0	
Biochemical recurrence (BCR)			0.160
Yes	3	1	
No	7	12	
Missing	1	0	
Clinical Stage			0.330
T2a	1	3	
T2c	6	9	
T3a	1	0	
T3b	3	1	
Surgical margins			0.480
Yes	1	0	
No	8	11	
Missing	2	2	
Family history of prostate cancer			0.830
No	3	5	
Yes	3	3	
Missing	5	5	

^1^*p*-Values were obtained from Student’s *t*, chi-square, or Fisher exact tests. PSA, prostate-specific antigen.

**Table 2:** Differentially methylated genes between aggressive (high-risk) and indolent (low-risk) prostate tumors in Puerto Rican men with prostate cancer.

Probe ID	Gene Symbol	GeneBody	Chr	Position	*p*-Value	Delta β-Value	Mean (Low-Risk)	Mean (High-Risk)
cg22030684	*COL9A2*	Body	1	40,781,708	0.000067	−0.213457	0.449935	0.236478
cg25161377	*FAM179A*	Body	2	29,237,783	0.000126	−0.221753	0.654878	0.433124
cg15218485	*RREB1*	Body	6	7,201,665	0.000899	0.220195	0.238283	0.458478
cg24163360	*SLC17A2*	TSS1500	6	25,931,557	0.000087	−0.218759	0.685592	0.466833
cg25641223	*PDE10A*	Body	6	165,747,945	0.000104	−0.200032	0.640184	0.440152
cg11747142	*FAM71F2*	TSS200	7	128,312,331	0.00054	0.229507	0.477201	0.706708
cg17983571	*JMJD1C*	Body	10	65,186,953	0.000427	0.23785	0.470854	0.708704
cg05363118	*PLEKHS1*	5′UTR	10	115,523,310	0.000081	−0.206065	0.589143	0.383078
cg05258834	*TNNI2*	Body	11	1,862,477	0.000455	−0.24573	0.648741	0.403012
cg21359838	*OR51A4*	TSS1500	11	4,969,708	0.000186	−0.212002	0.572201	0.360198
cg25921194	*RNF169*	Body	11	74,492,567	0.000161	−0.24419	0.624767	0.380577
cg19092163	*SPNS2*	Body	17	4,403,417	0.00075	−0.231457	0.433052	0.201596
cg15002904	*ADAMTSL5*	Body	19	1,510,692	0.000368	−0.242087	0.496689	0.254602
cg17713488	*COL5A3*	Body	19	10,077,935	0.000157	0.360569	0.405099	0.765668
cg22669123	*CYP4F12*	TSS1500	19	15,782,644	0.000677	−0.215233	0.627928	0.412695
cg09154639	*RAE1*	TSS1500	20	55,925,570	0.000905	0.226258	0.561288	0.787546
cg14539730	*GABRQ*	Body	X	151,809,946	0.000227	0.389047	0.362807	0.751854

**Table 3:** Ancestry proportions in the study cohort of 23 Puerto Rican men with prostate cancer.

Ancestral Population	Average	SD	Maximum	Minimum
African	0.2408	0.2256	0.8576	0.0274
European	0.6420	0.2181	0.9716	0.0938
Indigenous American	0.1173	0.0897	0.2516	0

SD: standard deviation.

## Data Availability

The data presented in this study are available on request from the corresponding author. The data are not publicly available due to privacy of participants.

## Supplementary Materials

The following are available online at https://www.mdpi.com/1422-0067/22/2/733/s1: Table S1. Differentially methylated CpG sites between tumor and adjacent normal prostate tissues

## Abbreviations

PCa	Prostate cancer
DRC	DNA repair capacity
NER	Nucleotide excision repair
IRB	Institutional Review Board
DMR	Differentially methylated region
PR	Puerto Rico
H/L	Hispanic Latino
AA	African American
NHW	Non-Hispanic White
PCA	Principal component analysis
AIMS	Ancestry informative markers
QA	Quality assurance
QC	Quality control
FFPE	Formalin-fixed paraffin-embedded
SNPs	Single-nucleotide polymorphisms
TCGA	The Cancer Genome Atlas
FDR	False discovery rate
PCR	Polymerase chain reaction
PHSU	Ponce Health Sciences University
PRI	Ponce Research Institute
PRBB	Puerto Rico Biobank
HR	Homologous recombination
PARP1	Poly (ADP-Ribose) polymerase-1
BRCA1	Breast cancer type 1 susceptibility protein
BRCA2	Breast cancer type 2 susceptibility protein
DSBs	DNA double-strand breaks
*AOX1*	*Aldehyde Oxidase 1*
*RREB1*	*Ras Responsive Element Binding Protein 1*
*FAM71F2*	*Family with Sequence Similarity 71 F2*
*JMJD1C*	*Jumonji Domain Containing 1C*
*COL5A3*	*Collagen Type V Alpha 3 Chain*
*RAE1*	*Ribonucleic Acid Export 1*
*GABRQ*	*Gamma-Aminobutyric Acid Type A Receptor Subunit Theta*
*COL9A2*	*Collagen Type IX Alpha 2 Chain*
*FAM179A*	*TOG Array Regulator of Axonemal Microtubules 2*
*SLC17A2*	*Solute Carrier Family 17 Member 2*
*PDE10*	*Phosphodiesterase 10*
*PLEKHS1*	*Pleckstrin Homology Domain Containing S1*
*TNNI2*	*Troponin I2, Fast Skeletal Type*
*OR51A4*	*Olfactory Receptor Family 51 Subfamily A Member 4*
*RNF169*	*Ring Finger Protein 169*
*SPNS2*	*Sphingolipid Transporter 2*
*ADAMTSL5*	*ADAMTS Like 5*
*CYP4F12*	*Cytochrome P450 Family 4 Subfamily F Member 12*
*MDC1*	*Mediator of DNA Damage Checkpoint 1*
*PARP*	*Poly(ADP-Ribose) Polymerase*
*BRCA1*	*BRCA1 DNA Repair Associated*
*BRCA2*	*BRCA2 DNA Repair Associated*
