# Supplementary material for: Differential DNA Methylation in Prostate Tumors from Puerto Rican Men
Source: Int J Mol Sci. 2021 Jan 13;22(2):733. doi: 10.3390/ijms22020733 (PMC7828429; doi:10.3390/ijms22020733)
Supplement: Supplementary file 1 [file ijms-22-00733-s001.pdf]

**Supplemental Table 1:** Differentially methylated CpG sites between tumor and adjacent normal prostate tissues

| ProbeId | Gene Symbol | Gene Body | # of probes | Chr | Position | q-value | Δβ-value | Mean (Normal) | Mean (Tumor) |
|---|---|---|---|---|---|---|---|---|---|
| cg27395066 | ACBD4 | 3'UTR;3'UTR;3'UTR;3'UTR | 2 | 17 | 43221220 | 0.00022422 | 0.312254 | 0.37267 | 0.684923 |
| cg00677574 | ACBD4 | 3'UTRUTR | | 17 | 43221327 | 0.00015868 | 0.317911 | 0.255584 | 0.573494 |
| cg11659501 | ACSS3 | TSS200 | 4 | 12 | 81471678 | 0.00031521 | 0.319392 | 0.236728 | 0.556119 |
| cg23007705 | ACSS3 | TSS200 | | 12 | 81471681 | 0.0002601 | 0.321452 | 0.280268 | 0.60172 |
| cg13273396 | ACSS3 | 1stExon;5'UTR | | 12 | 81471867 | 0.00031146 | 0.318743 | 0.305271 | 0.624014 |
| cg10789281 | ACSS3 | 1stExon;5'UTR | | 12 | 81471882 | 0.00040705 | 0.304475 | 0.216886 | 0.521361 |
| cg00905687 | ADAMTS12 | 1stExon | 4 | 5 | 33891893 | 0.0001719 | 0.304883 | 0.27945 | 0.584333 |
| cg24744455 | ADAMTS12 | 1stExon;5'UTR | | 5 | 33892083 | 0.0001183 | 0.389836 | 0.25816 | 0.647996 |
| cg26573704 | ADAMTS12 | TSS200 | | 5 | 33892223 | 0.00047726 | 0.304221 | 0.235327 | 0.539548 |
| cg21264391 | ADAMTS12 | TSS200 | | 5 | 33892231 | 0.00083979 | 0.307322 | 0.237448 | 0.544771 |
| cg15798800 | ALDH1L1 | TSS2000;1stExon;5'UTR;Body | 2 | 3 | 125899832 | 0.00010991 | 0.316987 | 0.194867 | 0.511854 |
| cg11022432 | ALDH1L1 | TSS1500 | | 3 | 125899878 | 0.00015618 | 0.344794 | 0.232931 | 0.577726 |
| cg21981270 | ALOX5 | Body | 4 | 10 | 45914525 | 0.00014194 | 0.365118 | 0.250481 | 0.615599 |
| cg16831085 | ALOX5 | Body | | 10 | 45914645 | 0.0001781 | 0.338594 | 0.219796 | 0.55839 |
| cg00675569 | ALOX5 | Body | | 10 | 45914840 | 0.00055143 | 0.300329 | 0.188766 | 0.489095 |
| cg09806262 | ALOX5 | Body | | 10 | 45914949 | 0.00017726 | 0.335103 | 0.252549 | 0.587652 |
| cg04380340 | AOX1 | TSS1500 | 9 | 2 | 201450506 | 0.00015069 | 0.366948 | 0.17512 | 0.542068 |
| cg13000082 | AOX1 | TSS1500 | | 2 | 201450527 | 0.00022224 | 0.310455 | 0.179668 | 0.490123 |
| cg09729613 | AOX1 | TSS200 | | 2 | 201450601 | 0.00025281 | 0.340351 | 0.16812 | 0.508471 |
| cg08952506 | AOX1 | TSS200 | | 2 | 201450610 | 0.00012306 | 0.364986 | 0.186797 | 0.551782 |
| cg02657832 | AOX1 | TSS200 | | 2 | 201450633 | 0.00014431 | 0.356014 | 0.198995 | 0.555009 |
| cg02144933 | AOX1 | TSS200 | | 2 | 201450690 | 0.00014141 | 0.378559 | 0.186649 | 0.565208 |
| cg12627583 | AOX1 | 1stExon;5'UTR | | 2 | 201450731 | 0.00019972 | 0.363519 | 0.302708 | 0.666227 |
| cg13875120 | AOX1 | 1stExon;5'UTR | | 2 | 201450743 | 0.00011842 | 0.445405 | 0.304077 | 0.749482 |
| cg14383422 | AOX1 | Body | | 2 | 201450962 | 0.00023561 | 0.349311 | 0.428918 | 0.778229 |
| cg00577935 | APC | TSS1500;5'UTR;TSS1500 | 9 | 5 | 112073348 | 0.00034312 | 0.348485 | 0.181574 | 0.53006 |

| | | | | | | | | | |
|---|---|---|---|---|---|---|---|---|---|
| cg08571859 | APC | TSS1500;5'UTR;TSS1500 | | 5 | 112073350 | 0.00013098 | 0.350413 | 0.196455 | 0.546869 |
| cg14511739 | APC | TSS200;TSS200;5'UTR | | 5 | 112073373 | 0.00035008 | 0.358942 | 0.212905 | 0.571846 |
| cg22035501 | APC | TSS200;TSS200;5'UTR | | 5 | 112073398 | 0.00037244 | 0.367056 | 0.200599 | 0.567655 |
| cg11613015 | APC | TSS200;TSS200;5'UTR | | 5 | 112073406 | 0.00023684 | 0.387485 | 0.244719 | 0.632204 |
| cg14479889 | APC | TSS200;TSS200;5'UTR | | 5 | 112073426 | 0.0001019 | 0.443098 | 0.232895 | 0.675993 |
| cg16970232 | APC | TSS200;TSS200;5'UTR | | 5 | 112073433 | 0.00011805 | 0.415827 | 0.210076 | 0.625903 |
| cg03667968 | APC | TSS200;TSS200;5'UTR | | 5 | 112073438 | 0.00013098 | 0.442097 | 0.248338 | 0.690435 |
| cg20311501 | APC | TSS200;TSS200;5'UTR | | 5 | 112073502 | 0.00013128 | 0.334659 | 0.24673 | 0.581389 |
| cg02786012 | ARL9 | 5'UTR;1stExon | 4 | 4 | 57371589 | 0.00014663 | 0.361728 | 0.204116 | 0.565844 |
| cg06922635 | ARL9 | 5'UTR;1stExon | | 4 | 57371607 | 0.0002717 | 0.335842 | 0.228657 | 0.564499 |
| cg15846316 | ARL9 | 5'UTR | | 4 | 57371719 | 0.00022548 | 0.348676 | 0.143457 | 0.492133 |
| cg04528060 | ARL9 | 5'UTR | | 4 | 57371868 | 0.00014598 | 0.320011 | 0.113041 | 0.433052 |
| cg17852104 | BHLHB9 | 5'UTR; | 3 | X | 102000694 | 0.00029373 | 0.306912 | 0.365666 | 0.672578 |
| cg09789426 | BHLHB9 | 5'UTR; | | X | 102000698 | 0.00018677 | 0.315975 | 0.307778 | 0.623753 |
| cg04039555 | BHLHB9 | TSS200;5'UTR;5'UTR; | | X | 102000746 | 0.00182599 | 0.309769 | 0.196077 | 0.505846 |
| cg04542030 | BOLA3 | TSS1500;TSS1500 | 2 | 2 | 74375469 | 9.67E-05 | 0.323151 | 0.232424 | 0.555574 |
| cg02051239 | BOLA3 | TSS1500;TSS1500 | | 2 | 74375507 | 0.00015584 | 0.32866 | 0.215953 | 0.544613 |
| cg04986616 | C11orf95 | TSS1500 | 3 | 11 | 63537378 | 0.0001183 | 0.385347 | 0.288985 | 0.674332 |
| cg09907758 | C11orf95 | TSS1500 | | 11 | 63537459 | 0.00015582 | 0.339216 | 0.344513 | 0.683728 |
| cg11686923 | C11orf95 | TSS1500 | | 11 | 63537473 | 0.00014918 | 0.33446 | 0.260061 | 0.594521 |
| cg13958426 | C1orf114 | 1stExon;5'UTR | 4 | 1 | 169396637 | 0.00053067 | 0.3052 | 0.190717 | 0.495918 |
| cg00002719 | C1orf114 | TSS200 | | 1 | 169396706 | 0.00042278 | 0.357403 | 0.245879 | 0.603282 |
| cg08104202 | C1orf114 | TSS200 | | 1 | 169396712 | 0.00051046 | 0.337702 | 0.22611 | 0.563812 |
| cg23818870 | C1orf114 | TSS200 | | 1 | 169396785 | 0.00014755 | 0.339276 | 0.232213 | 0.571489 |
| cg10426464 | C2CD4B | TSS200 | 2 | 15 | 62457529 | 0.00021669 | 0.342036 | 0.224946 | 0.566982 |
| cg08472125 | C2CD4B | TSS200 | | 15 | 62457537 | 0.00029441 | 0.330351 | 0.263821 | 0.594172 |
| cg10168635 | C2orf88 | TSS1500;5'UTR;TSS1500;5'UTR | 2 | 2 | 191044980 | 0.00012365 | 0.307245 | 0.269935 | 0.57718 |
| cg11448068 | C2orf88 | TSS1500;5'UTR;TSS1500;5'UTR | | 2 | 191045026 | 0.0001167 | 0.343871 | 0.196354 | 0.540225 |
| cg00604840 | C5orf4 | 5'UTR;1stExon | 4 | 5 | 154230173 | 0.0001008 | 0.370954 | 0.244483 | 0.615437 |

| | | | | | | | | | |
|---|---|---|---|---|---|---|---|---|---|
| cg18188717 | C5orf4 | TSS200 | | 5 | 154230223 | 0.00010938 | 0.353325 | 0.25044 | 0.603764 |
| cg10257049 | C5orf4 | TSS200 | | 5 | 154230308 | 0.00012287 | 0.332343 | 0.297476 | 0.629819 |
| cg18260397 | C5orf4 | TSS1500 | | 5 | 154230438 | 0.00012306 | 0.308226 | 0.211019 | 0.519245 |
| cg21088420 | C5orf49 | Body | 3 | 5 | 7849836 | 0.00010886 | 0.332535 | 0.270445 | 0.60298 |
| cg25214789 | C5orf49 | Body | | 5 | 7850070 | 0.00018525 | 0.324669 | 0.213932 | 0.538601 |
| cg12539796 | C5orf49 | Body | | 5 | 7850203 | 0.00057368 | 0.310047 | 0.358449 | 0.668496 |
| cg05355225 | CA3 | TSS1500 | 3 | 8 | 86350568 | 0.0003813 | 0.350947 | 0.288779 | 0.639726 |
| cg18674980 | CA3 | TSS1500 | | 8 | 86350581 | 0.00025459 | 0.335992 | 0.304629 | 0.640621 |
| cg07560510 | CA3 | TSS1500 | | 8 | 86350592 | 0.00023911 | 0.353458 | 0.258359 | 0.611817 |
| cg20265803 | CCDC8 | TSS200 | 2 | 19 | 46917036 | 0.00016324 | 0.318709 | 0.279405 | 0.598114 |
| cg03576469 | CCDC8 | TSS200 | | 19 | 46917061 | 0.00010614 | 0.354532 | 0.21135 | 0.565882 |
| cg26057751 | CD8A | Body;Body;Body;Body | 2 | 2 | 87016259 | 0.00013971 | 0.335166 | 0.373639 | 0.708804 |
| cg07152196 | CD8A | Body;Body;Body;Body | | 2 | 87016436 | 0.00013423 | 0.309979 | 0.189231 | 0.49921 |
| cg21929477 | CHN2 | TSS1500;5'UTR;5'UTR | 2 | 7 | 29185545 | 0.00012489 | 0.326751 | 0.247 | 0.573752 |
| cg12334180 | CHN2 | TSS1500;5'UTR;5'UTR | | 7 | 29185839 | 0.0001318 | 0.301048 | 0.237873 | 0.538922 |
| cg01940855 | CHST11 | Body | 2 | 12 | 104852355 | 0.00014725 | 0.316546 | 0.158381 | 0.474927 |
| cg23855505 | CHST11 | Body | | 12 | 104852439 | 0.00031979 | 0.316101 | 0.207934 | 0.524035 |
| cg06284231 | CLEC14A | 1stExon | 3 | 14 | 38724255 | 0.000159 | 0.334583 | 0.287559 | 0.622142 |
| cg25292550 | CLEC14A | 1stExon | | 14 | 38724646 | 0.00018322 | 0.325035 | 0.281302 | 0.606337 |
| cg16404157 | CLEC14A | 1stExon | | 14 | 38724648 | 0.00019804 | 0.301744 | 0.302385 | 0.604129 |
| cg07285673 | CLIP4 | TSS1500 | 4 | 2 | 29337984 | 0.00017453 | 0.31142 | 0.24887 | 0.56029 |
| cg09695033 | CLIP4 | TSS1500 | | 2 | 29337988 | 0.00018477 | 0.303963 | 0.206711 | 0.510674 |
| cg25737323 | CLIP4 | TSS1500 | | 2 | 29338100 | 0.00068285 | 0.301948 | 0.222125 | 0.524073 |
| cg13257636 | CLIP4 | TSS200 | | 2 | 29338109 | 0.0004104 | 0.336903 | 0.249227 | 0.58613 |
| cg18107562 | CPVL | 1stExon;TSS200;5'UTR;5'UTR | 2 | 7 | 29185963 | 0.00048048 | 0.300998 | 0.245975 | 0.546973 |
| cg22187722 | CPVL | 5'UTR;1stExon;5'UTR | | 7 | 29185965 | 0.00031161 | 0.325997 | 0.290849 | 0.616845 |
| cg26537639 | CYBA | 1stExon | 2 | 16 | 88717374 | 0.00030116 | 0.304318 | 0.302515 | 0.606833 |
| cg04879832 | CYBA | 1stExon;5'UTR | | 16 | 88717456 | 0.00027598 | 0.374895 | 0.27146 | 0.646355 |
| cg02916102 | CYP11A1 | 1stExon;Body;5'UTR | 2 | 15 | 74658244 | 0.00017204 | 0.316705 | 0.183509 | 0.500215 |

| | | | | | | | | | |
|---|---|---|---|---|---|---|---|---|---|
| cg16332610 | CYP11A1 | 1stExon;Body;5'UTR | | 15 | 74658547 | 0.000206 | 0.343996 | 0.260629 | 0.604625 |
| cg06857304 | CYP26C1 | Body | 2 | 10 | 94826314 | 0.00022721 | 0.318439 | 0.357187 | 0.675626 |
| cg02466572 | CYP26C1 | Body | | 10 | 94826319 | 0.00044771 | 0.307283 | 0.309285 | 0.616568 |
| cg03460682 | CYP27A1 | TSS200 | 5 | 2 | 219646316 | 0.00011794 | 0.323631 | 0.117635 | 0.441267 |
| cg22811307 | CYP27A1 | TSS200 | | 2 | 219646318 | 0.00011794 | 0.314871 | 0.116054 | 0.430926 |
| cg08111152 | CYP27A1 | TSS200 | | 2 | 219646331 | 8.38E-05 | 0.305968 | 0.135053 | 0.441021 |
| cg10147666 | CYP27A1 | 5'UTR;1stExon | | 2 | 219646481 | 9.67E-05 | 0.42119 | 0.214238 | 0.635428 |
| cg14553243 | CYP27A1 | 5'UTR;1stExon | | 2 | 219646657 | 8.85E-05 | 0.391133 | 0.212974 | 0.604107 |
| cg01532436 | CYTH2 | 3'UTR;3'UTR | 2 | 19 | 48983942 | 0.00011911 | 0.317608 | 0.281937 | 0.599545 |
| cg02590401 | CYTH2 | 3'UTR;3'UTR | | 19 | 48984071 | 0.00011794 | 0.34291 | 0.279429 | 0.622339 |
| cg04760071 | DNAH8 | TSS200 | 2 | 6 | 38682982 | 0.00020027 | 0.320433 | 0.26551 | 0.585943 |
| cg08102185 | DNAH8 | TSS200 | | 6 | 38682995 | 0.00027989 | 0.304767 | 0.25701 | 0.561778 |
| cg19295951 | DUOX1 | TSS200;TSS200;1stExon;5'UTR | 6 | 15 | 45421998 | 0.00088326 | 0.358635 | 0.206129 | 0.564764 |
| cg08328777 | DUOX1 | TSS200;TSS200;1stExon;5'UTR | | 15 | 45422004 | 0.00068445 | 0.389594 | 0.224338 | 0.613932 |
| cg24505618 | DUOX1 | TSS200;TSS200;1stExon;5'UTR | | 15 | 45422054 | 0.00066029 | 0.350828 | 0.190559 | 0.541387 |
| cg05804220 | DUOX1 | TSS200;TSS200;TSS200 | | 15 | 45422062 | 0.00066446 | 0.381629 | 0.222552 | 0.604181 |
| cg11230298 | DUOX1 | TSS200;TSS200;1stExon; | | 15 | 45422066 | 0.00082319 | 0.343501 | 0.196302 | 0.539803 |
| cg06646705 | DUOX1 | TSS200;TSS200;TSS200; | | 15 | 45422081 | 0.00154233 | 0.30014 | 0.196205 | 0.496346 |
| cg04138112 | DUSP27 | Body | 2 | 1 | 167090618 | 0.0004743 | 0.323922 | 0.282253 | 0.606175 |
| cg17104258 | DUSP27 | Body | | 1 | 167090646 | 0.00040867 | 0.313019 | 0.20849 | 0.521509 |
| cg21489390 | ELF4 | 5'UTR;5'UTR | 2 | X | 129243806 | 0.00018217 | 0.329557 | 0.214613 | 0.54417 |
| cg06428055 | ELF4 | 5'UTR;5'UTR | | X | 129243872 | 0.00015023 | 0.362374 | 0.207083 | 0.569458 |
| cg21113740 | ELTD1 | 5'UTR;1stExon | 3 | 1 | 79472343 | 0.00023251 | 0.355743 | 0.291081 | 0.646825 |
| cg04360793 | ELTD1 | 5'UTR;1stExon | | 1 | 79472361 | 0.00025479 | 0.355996 | 0.37974 | 0.735736 |
| cg15084543 | ELTD1 | 5'UTR;1stExon | | 1 | 79472408 | 0.00056385 | 0.308126 | 0.317414 | 0.62554 |
| cg07641160 | FAM162B | 1stExon;5'UTR | 2 | 6 | 117086850 | 0.00049929 | 0.314199 | 0.210497 | 0.524696 |
| cg13631916 | FAM162B | TSS200 | | 6 | 117086931 | 0.00033912 | 0.317469 | 0.172185 | 0.489654 |
| cg13319975 | FBXO30 | TSS1500 | 4 | 6 | 146136371 | 0.00024025 | 0.315023 | 0.214342 | 0.529364 |
| cg05347473 | FBXO30 | TSS1500 | | 6 | 146136440 | 0.00031571 | 0.370531 | 0.322355 | 0.692886 |

| | | | | | | | | | |
|---|---|---|---|---|---|---|---|---|---|
| cg23095615 | FBXO30 | TSS1500 | | 6 | 146136563 | 0.00011746 | 0.383493 | 0.259746 | 0.64324 |
| cg09094393 | FBXO30 | TSS1500 | | 6 | 146136749 | 0.0001019 | 0.389679 | 0.265196 | 0.654875 |
| cg06711837 | FGF13 | Body; | 3 | X | 137792814 | 0.00014249 | 0.322944 | 0.318591 | 0.641535 |
| cg06525551 | FGF13 | Body; | | X | 137792939 | 0.00021246 | 0.30106 | 0.208532 | 0.509592 |
| cg05196026 | FGF13 | Body;1stExon;Body;Body | | X | 137793096 | 0.00023926 | 0.372539 | 0.318116 | 0.690656 |
| cg13123964 | FGFR1 | 5'UTR; | 2 | 8 | 38323238 | 0.00016894 | 0.312319 | 0.23403 | 0.546349 |
| cg00400221 | FGFR1 | 5'UTR; | | 8 | 38323291 | 0.00011108 | 0.365908 | 0.225014 | 0.590922 |
| cg11280896 | FJX1 | TSS1500 | 3 | 11 | 35639365 | 0.00021139 | 0.303405 | 0.244969 | 0.548374 |
| cg15536165 | FJX1 | TSS1500 | | 11 | 35639492 | 0.00018312 | 0.309531 | 0.213756 | 0.523287 |
| cg10160713 | FJX1 | TSS1500 | | 11 | 35639524 | 0.00019473 | 0.312229 | 0.15223 | 0.464458 |
| cg04448487 | GDAP1L1 | TSS200 | 2 | 20 | 42875777 | 0.00023609 | 0.362166 | 0.226159 | 0.588325 |
| cg09994356 | GDAP1L1 | TSS200 | | 20 | 42875779 | 0.000735 | 0.351771 | 0.185202 | 0.536973 |
| cg02288341 | GFRA3 | 1stExon;5'UTR | 4 | 5 | 137610226 | 0.00035571 | 0.316026 | 0.24145 | 0.557476 |
| cg23511432 | GFRA3 | TSS200 | | 5 | 137610255 | 0.00061219 | 0.313667 | 0.199456 | 0.513124 |
| cg09350274 | GFRA3 | TSS200 | | 5 | 137610396 | 0.0005309 | 0.31611 | 0.243663 | 0.559773 |
| cg15951557 | GFRA3 | TSS200 | | 5 | 137610421 | 0.00021899 | 0.319044 | 0.204434 | 0.523478 |
| cg24818939 | GPRASP1 | TSS200;TSS200;TSS200 | 2 | X | 101906109 | 0.00028912 | 0.37689 | 0.176412 | 0.553303 |
| cg15579650 | GPRASP1 | TSS200;TSS200;TSS200 | | X | 101906119 | 0.00031207 | 0.354369 | 0.144275 | 0.498644 |
| cg13081720 | GPX3 | TSS200 | 2 | 5 | 150399890 | 0.00028824 | 0.307401 | 0.236009 | 0.54341 |
| cg08891071 | GPX3 | TSS200 | | 5 | 150399909 | 0.00051499 | 0.331118 | 0.252037 | 0.583155 |
| cg21511941 | GRAMD2 | Body | 2 | 15 | 72489609 | 0.00012306 | 0.365086 | 0.372217 | 0.737303 |
| cg04098270 | GRAMD2 | Body | | 15 | 72489686 | 0.00023608 | 0.328566 | 0.22226 | 0.550826 |
| cg09038676 | GSTP1 | Body | 2 | 11 | 67351608 | 0.00011667 | 0.358899 | 0.226985 | 0.585884 |
| cg11566244 | GSTP1 | Body | | 11 | 67351786 | 0.00024916 | 0.309843 | 0.304175 | 0.614018 |
| cg15924217 | GSX2 | TSS1500 | 4 | 4 | 54965684 | 0.00035076 | 0.306449 | 0.194286 | 0.500734 |
| cg14686113 | GSX2 | TSS1500 | | 4 | 54965829 | 0.00065031 | 0.303892 | 0.172232 | 0.476124 |
| cg13506653 | GSX2 | TSS1500 | | 4 | 54965863 | 0.0004546 | 0.318206 | 0.23776 | 0.555965 |
| cg21905630 | GSX2 | TSS1500 | | 4 | 54965904 | 0.00039329 | 0.325667 | 0.234053 | 0.55972 |
| cg26360792 | HAAO | TSS1500 | 2 | 2 | 43019997 | 0.00025842 | 0.340756 | 0.23981 | 0.580565 |

| | | | | | | | | | |
|---|---|---|---|---|---|---|---|---|---|
| cg27299406 | HAAO | TSS1500 | | 2 | 43020013 | 0.00027273 | 0.336955 | 0.200949 | 0.537904 |
| cg14449180 | HCG4P6 | TSS1500 | 4 | 6 | 29894619 | 0.00022352 | 0.360473 | 0.394197 | 0.75467 |
| cg03995122 | HCG4P6 | TSS1500 | | 6 | 29894642 | 0.00016336 | 0.374519 | 0.308798 | 0.683318 |
| cg08231349 | HCG4P6 | TSS1500 | | 6 | 29894644 | 0.00020027 | 0.337403 | 0.389785 | 0.727188 |
| cg00409917 | HCG4P6 | TSS1500 | | 6 | 29894679 | 0.00022761 | 0.366211 | 0.362902 | 0.729113 |
| cg20430847 | HES5 | TSS200 | 5 | 1 | 2461820 | 0.0001529 | 0.325621 | 0.2326 | 0.55822 |
| cg09827752 | HES5 | TSS200 | | 1 | 2461823 | 0.00017381 | 0.332412 | 0.321058 | 0.65347 |
| cg17755964 | HES5 | TSS200 | | 1 | 2461834 | 0.00021109 | 0.306769 | 0.307846 | 0.614615 |
| cg18040786 | HES5 | TSS200 | | 1 | 2461875 | 0.00013334 | 0.327757 | 0.199021 | 0.526778 |
| cg06839900 | HES5 | TSS1500 | | 1 | 2461900 | 0.00012287 | 0.317436 | 0.225524 | 0.54296 |
| cg19320816 | HFE | Body; | 2 | 6 | 26087776 | 0.00038057 | 0.320726 | 0.167524 | 0.48825 |
| cg04156555 | HFE | Body; | | 6 | 26087816 | 0.00024486 | 0.309317 | 0.180742 | 0.490059 |
| cg14117138 | HIF3A | TSS1500 | 3 | 19 | 46800085 | 0.00013667 | 0.380994 | 0.286651 | 0.667645 |
| cg21544503 | HIF3A | TSS200;Body | | 19 | 46800131 | 0.00021885 | 0.342622 | 0.289825 | 0.632446 |
| cg14088357 | HIF3A | TSS1500;1stExon | | 19 | 46800338 | 0.00013098 | 0.359097 | 0.264547 | 0.623643 |
| cg26751972 | HLA-F | Body;Body;Body | 2 | 6 | 29692000 | 9.56E-05 | 0.377977 | 0.34873 | 0.726707 |
| cg07016276 | HLA-F | Body;Body;Body | | 6 | 29692009 | 0.0001538 | 0.303209 | 0.169754 | 0.472963 |
| cg09003023 | HLA-J | Body;Body | 14 | 6 | 29974253 | 0.00014598 | 0.331047 | 0.370186 | 0.701233 |
| cg08879910 | HLA-J | Body;Body | | 6 | 29974319 | 0.00012789 | 0.422116 | 0.372834 | 0.794949 |
| cg08163199 | HLA-J | Body;Body | | 6 | 29974858 | 0.00017438 | 0.380048 | 0.275891 | 0.655938 |
| cg25318809 | HLA-J | Body;Body | | 6 | 29974863 | 0.00019452 | 0.37163 | 0.275833 | 0.647463 |
| cg14781281 | HLA-J | Body;Body | | 6 | 29974868 | 0.00019918 | 0.361047 | 0.236456 | 0.597503 |
| cg08325845 | HLA-J | Body;Body | | 6 | 29974886 | 0.00013533 | 0.383275 | 0.296015 | 0.67929 |
| cg18713646 | HLA-J | Body;Body | | 6 | 29974952 | 0.00013098 | 0.300944 | 0.232952 | 0.533896 |
| cg16794576 | HLA-J | Body;Body | | 6 | 29974971 | 0.0001183 | 0.319333 | 0.18579 | 0.505122 |
| cg12976581 | HLA-J | Body;Body | | 6 | 29975032 | 0.00011056 | 0.370217 | 0.253774 | 0.623992 |
| cg14432143 | HLA-J | Body;Body | | 6 | 29975061 | 8.85E-05 | 0.339647 | 0.18529 | 0.524938 |
| cg21330423 | HLA-J | Body;Body | | 6 | 29975068 | 0.00014141 | 0.368362 | 0.223483 | 0.591845 |
| cg09659004 | HLA-J | Body;Body | | 6 | 29975078 | 0.00014337 | 0.32907 | 0.190613 | 0.519684 |

| | | | | | | | | | |
|---|---|---|---|---|---|---|---|---|---|
| cg05998089 | HLA-J | Body;Body | | 6 | 29975141 | 8.85E-05 | 0.309006 | 0.191474 | 0.50048 |
| cg08598483 | HLA-J | Body;Body | | 6 | 29975145 | 0.00021011 | 0.302342 | 0.19891 | 0.501252 |
| cg21899596 | HOPX | 1stExon;5'UTR; | 2 | 4 | 57522493 | 0.00023011 | 0.36361 | 0.318562 | 0.682172 |
| cg24852548 | HOPX | 1stExon;5'UTR | | 4 | 57522632 | 0.0001875 | 0.320425 | 0.218564 | 0.538989 |
| cg23618344 | HOXC4 | 5'UTR;Body;Body;Body | 10 | 12 | 54423428 | 0.0001764 | 0.327443 | 0.262759 | 0.590202 |
| cg07687119 | HOXC4 | 5'UTR;Body;Body;Body | | 12 | 54423549 | 0.00016727 | 0.344455 | 0.309514 | 0.653969 |
| cg18054172 | HOXC4 | 5'UTR;Body | | 12 | 54424902 | 0.00012306 | 0.316827 | 0.283318 | 0.600144 |
| cg10005224 | HOXC4 | 5'UTR;Body | | 12 | 54424964 | 0.0001916 | 0.330704 | 0.306564 | 0.637268 |
| cg18843682 | HOXC4 | 5'UTR;Body | | 12 | 54425156 | 0.00015066 | 0.300983 | 0.313176 | 0.614159 |
| cg03923561 | HOXC4 | 5'UTR;TSS1500 | | 12 | 54447220 | 0.00018127 | 0.359916 | 0.270388 | 0.630304 |
| cg02264990 | HOXC4 | 5'UTR;TSS1500 | | 12 | 54447243 | 0.00030719 | 0.32069 | 0.174868 | 0.495557 |
| cg07266404 | HOXC4 | 5'UTR;TSS200 | | 12 | 54447584 | 0.00012259 | 0.362258 | 0.262039 | 0.624298 |
| cg15894722 | HOXC4 | 5'UTR;TSS200 | | 12 | 54447596 | 0.0002045 | 0.340454 | 0.293578 | 0.634031 |
| cg20435403 | HOXC4 | TSS200;5'UTR | | 12 | 54447632 | 0.00034004 | 0.343859 | 0.254831 | 0.598691 |
| cg15071854 | HPDL | 1stExon;5'UTR | 2 | 1 | 45792688 | 0.00185052 | 0.317442 | 0.249994 | 0.567436 |
| cg12178578 | HPDL | 1stExon;5'UTR | | 1 | 45792714 | 0.00205776 | 0.318894 | 0.218773 | 0.537667 |
| cg14820199 | HS3ST1 | 5'UTR | 2 | 4 | 11429415 | 0.00013333 | 0.323819 | 0.215876 | 0.539695 |
| cg24029517 | HS3ST1 | 5'UTR | | 4 | 11429531 | 0.00030187 | 0.300505 | 0.243373 | 0.543879 |
| cg22774088 | JSRP1 | Body | 2 | 19 | 2253398 | 0.00025871 | 0.318506 | 0.288958 | 0.607464 |
| cg18128164 | JSRP1 | Body | | 19 | 2253492 | 0.00012961 | 0.301561 | 0.264718 | 0.566279 |
| cg14711997 | KCNH2 | 5'UTR;Body;Body;1stExon | 5 | 7 | 150652864 | 0.00013533 | 0.389383 | 0.37564 | 0.765023 |
| cg20883831 | KCNH2 | TSS200;Body;Body | | 7 | 150652948 | 0.00036764 | 0.317769 | 0.210443 | 0.528212 |
| cg15472092 | KCNH2 | TSS200;Body;Body | | 7 | 150653079 | 0.00012306 | 0.372243 | 0.252418 | 0.624661 |
| cg18296036 | KCNH2 | Body;Body;TSS1500 | | 7 | 150653115 | 0.00020354 | 0.333849 | 0.236644 | 0.570493 |
| cg24830730 | KCNH2 | Body;Body;TSS1500 | | 7 | 150653195 | 0.00014625 | 0.370288 | 0.282976 | 0.653264 |
| cg02822259 | KCNH6 | Body;Body | 2 | 17 | 61615504 | 0.00013971 | 0.33045 | 0.260367 | 0.590817 |
| cg05521586 | KCNH6 | Body;Body;Body;Body | | 17 | 61615566 | 0.00013971 | 0.30705 | 0.365429 | 0.672479 |
| cg04117869 | KLHL1 | 1stExon;1stExon;Body | 2 | 13 | 70681815 | 0.00026788 | 0.326398 | 0.277199 | 0.603598 |
| cg15197609 | KLHL1 | 1stExon;1stExon;5'UTR;;Body | | 13 | 70681838 | 0.0003305 | 0.304619 | 0.269383 | 0.574002 |

| | | | | | | | | | |
|---|---|---|---|---|---|---|---|---|---|
| cg01891172 | KLHL34 | TSS200 | 2 | X | 21676483 | 0.00101151 | 0.309294 | 0.254896 | 0.56419 |
| cg01828474 | KLHL34 | TSS200 | | X | 21676593 | 0.00061567 | 0.307816 | 0.278581 | 0.586397 |
| cg13490403 | LHX6 | Body;Body | 2 | 9 | 124982413 | 0.00031076 | 0.305544 | 0.28855 | 0.594094 |
| cg17930194 | LHX6 | Body;Body | | 9 | 124982834 | 0.00014252 | 0.309796 | 0.366746 | 0.676542 |
| cg05730916 | LINC01273 | TSS200 | 2 | 20 | 48789074 | 0.0001019 | 0.304795 | 0.19678 | 0.501575 |
| cg05499559 | LINC01273 | Body | | 20 | 48789176 | 0.00013759 | 0.310935 | 0.249147 | 0.560083 |
| cg20771240 | LOC100128239 | Body | 2 | 11 | 133906693 | 0.0001183 | 0.423195 | 0.275456 | 0.698651 |
| cg06769296 | LOC100128239 | Body | | 11 | 133906761 | 0.00011559 | 0.387523 | 0.291272 | 0.678795 |
| cg23200020 | LOC643719 | Body | 2 | 19 | 35068555 | 0.00071063 | 0.306119 | 0.225996 | 0.532115 |
| cg07599133 | LOC643719 | TSS200 | | 19 | 35068628 | 0.00064731 | 0.306971 | 0.238455 | 0.545426 |
| cg11909912 | MAPT | 5'UTR | 3 | 17 | 43974919 | 0.00012306 | 0.366953 | 0.278211 | 0.645164 |
| cg04382470 | MAPT | 5'UTR; | | 17 | 43974975 | 0.00015732 | 0.379082 | 0.264372 | 0.643454 |
| cg10224600 | MAPT | 5'UTR; | | 17 | 43975063 | 0.00052734 | 0.314288 | 0.322434 | 0.636722 |
| cg25568066 | MARCH3 | 3'UTR | | 5 | 126205045 | 0.00011532 | 0.346252 | 0.206646 | 0.552899 |
| cg06918474 | MARCH3 | 3'UTR | | 5 | 126205081 | 0.00014337 | 0.379809 | 0.25432 | 0.634129 |
| cg14173147 | MOBKL2B | 5'UTR | 3 | 9 | 27528300 | 0.00013334 | 0.341591 | 0.209441 | 0.551031 |
| cg21249376 | MOBKL2B | 5'UTR | | 9 | 27528432 | 0.00014252 | 0.321615 | 0.200228 | 0.521843 |
| cg22262168 | MOBKL2B | 5'UTR | | 9 | 27528999 | 0.00027331 | 0.30348 | 0.224591 | 0.528071 |
| cg13021619 | NKX2-6 | 1stExon | 6 | 8 | 23563859 | 0.00029731 | 0.321718 | 0.262395 | 0.584113 |
| cg14428146 | NKX2-6 | TSS200 | | 8 | 23563925 | 0.00039866 | 0.321951 | 0.235762 | 0.557713 |
| cg15854847 | NKX2-6 | TSS200 | | 8 | 23563970 | 0.00021724 | 0.328105 | 0.237191 | 0.565295 |
| cg22747746 | NKX2-6 | TSS1500 | | 8 | 23564193 | 0.00020688 | 0.357124 | 0.22706 | 0.584184 |
| cg10603004 | NKX2-6 | TSS1500 | | 8 | 23564294 | 0.00057595 | 0.304575 | 0.304059 | 0.608634 |
| cg17875555 | NKX2-6 | TSS1500 | | 8 | 23564490 | 0.00055183 | 0.380737 | 0.394891 | 0.775628 |
| cg23836455 | NOL3 | TSS200;TSS200;TSS200;TSS200 | 2 | 16 | 67204226 | 0.00026924 | 0.303755 | 0.195946 | 0.499701 |
| cg20439430 | NOL3 | TSS200;TSS200;TSS200;TSS200 | | 16 | 67204228 | 0.0004204 | 0.309147 | 0.189734 | 0.498881 |
| cg04850731 | NXPH4 | Body | 3 | 12 | 57618943 | 0.0001394 | 0.332735 | 0.42312 | 0.755856 |
| cg11505661 | NXPH4 | Body | | 12 | 57618965 | 0.00010938 | 0.317699 | 0.357676 | 0.675375 |
| cg22215815 | NXPH4 | Body | | 12 | 57619178 | 8.85E-05 | 0.375273 | 0.284754 | 0.660027 |

| | | | | | | | | | | |
|---|---|---|---|---|---|---|---|---|---|---|
| cg24771804 | ONECUT2 | Body | 2 | 18 | 55107755 | 0.0003067 | 0.307913 | 0.202162 | 0.510075 |
| cg00196827 | ONECUT2 | Body | | 18 | 55108068 | 0.00086801 | 0.313468 | 0.228975 | 0.542443 |
| cg08300419 | PACSIN3 | TSS1500 | 2 | 11 | 47208959 | 0.00016233 | 0.321434 | 0.267495 | 0.588929 |
| cg24779587 | PACSIN3 | TSS1500 | | 11 | 47209163 | 0.00011345 | 0.339976 | 0.257376 | 0.597351 |
| cg13687039 | PAQR9 | TSS1500;Body | 2 | 3 | 142683153 | 0.00046359 | 0.343253 | 0.276471 | 0.619724 |
| cg01879805 | PAQR9 | TSS1500;Body | | 3 | 142683164 | 0.00099594 | 0.314776 | 0.349194 | 0.66397 |
| cg23445461 | PCDHGA4 | Body; | 2 | 5 | 140864733 | 0.00023336 | 0.337289 | 0.252058 | 0.589347 |
| cg12145907 | PCDHGA4 | Body; | | 5 | 140864834 | 8.23E-05 | 0.32222 | 0.341631 | 0.663851 |
| cg18242030 | PDE4D | Body;Body;Body | 4 | 5 | 58335271 | 0.00054006 | 0.317866 | 0.202361 | 0.520226 |
| cg06768177 | PDE4D | TSS200;TSS200;Body; | | 5 | 58335472 | 0.00048771 | 0.370415 | 0.185393 | 0.555808 |
| cg01510198 | PDE4D | TSS200;TSS200;Body; | | 5 | 58335483 | 0.00063847 | 0.351248 | 0.184459 | 0.535707 |
| cg13894081 | PDE4D | TSS200;TSS200;Body; | | 5 | 58335508 | 0.00019901 | 0.378656 | 0.242417 | 0.621072 |
| cg19276365 | PDE4D | TSS200;TSS200;Body; | | 5 | 58335517 | 0.00017919 | 0.315228 | 0.213838 | 0.529067 |
| cg09329826 | PDZD4 | Body | 2 | X | 153094594 | 0.00020315 | 0.350564 | 0.34294 | 0.693504 |
| cg12763824 | PDZD4 | Body | | X | 153094947 | 0.00030253 | 0.302704 | 0.225768 | 0.528472 |
| cg07803474 | PODN | TSS200;TSS200;TSS200;TSS1500 | 2 | 1 | 53527551 | 0.00015673 | 0.319127 | 0.24035 | 0.559477 |
| cg04765848 | PODN | TSS1500 | | 1 | 53527576 | 0.00016786 | 0.304748 | 0.218817 | 0.523565 |
| cg14130039 | PPT2 | TSS200;TSS1500 | 2 | 6 | 32121225 | 0.0001615 | 0.332064 | 0.236094 | 0.568158 |
| cg05133205 | PPT2 | TSS200;TSS1500 | | 6 | 32121249 | 0.00035104 | 0.30757 | 0.237224 | 0.544794 |
| cg13164157 | PROM1 | 5'UTR;5'UTR | 8 | 4 | 16085180 | 0.00027901 | 0.319047 | 0.184692 | 0.503739 |
| cg18177979 | PROM1 | 5'UTR;5'UTR;1stExon | | 4 | 16085367 | 0.00010065 | 0.414844 | 0.201833 | 0.616677 |
| cg13709870 | PROM1 | 5'UTR;5'UTR;1stExon | | 4 | 16085546 | 0.0001915 | 0.308042 | 0.342511 | 0.650553 |
| cg14736058 | PROM1 | TSS200;5'UTR;1stExon | | 4 | 16085606 | 0.00010004 | 0.400412 | 0.308395 | 0.708807 |
| cg10630155 | PROM1 | TSS200;TSS200 | | 4 | 16085666 | 0.0001019 | 0.32224 | 0.313106 | 0.635346 |
| cg04203238 | PROM1 | TSS200;TSS200 | | 4 | 16085702 | 0.00014962 | 0.325073 | 0.342137 | 0.66721 |
| cg26260038 | PROM1 | TSS200;TSS200 | | 4 | 16085716 | 0.00011794 | 0.307811 | 0.370925 | 0.678736 |
| cg18802202 | PROM1 | TSS200;TSS200 | | 4 | 16085721 | 0.0001019 | 0.344823 | 0.288872 | 0.633694 |
| cg07910560 | PTPRN2 | Body;Body;Body | 9 | 7 | 157361396 | 0.0001183 | 0.310953 | 0.27406 | 0.585013 |
| cg13937162 | PTPRN2 | Body;Body;Body;Body;Body | | 7 | 157361545 | 0.00010991 | 0.369718 | 0.215936 | 0.585654 |

| | | | | | | | | | |
|---|---|---|---|---|---|---|---|---|---|
| cg00099393 | PTPRN2 | Body;Body;Body | | 7 | 157361621 | 0.00011725 | 0.30083 | 0.234674 | 0.535503 |
| cg04288257 | PTPRN2 | ExonBnd;ExonBnd;ExonBnd | | 7 | 157361639 | 0.00013334 | 0.318706 | 0.265636 | 0.584343 |
| cg11318054 | PTPRN2 | Body;Body;Body | | 7 | 157361692 | 0.0001183 | 0.391938 | 0.237012 | 0.62895 |
| cg21606115 | PTPRN2 | Body;Body;Body | | 7 | 157361752 | 9.49E-05 | 0.357459 | 0.255713 | 0.613173 |
| cg08022244 | PTPRN2 | Body;Body;Body | | 7 | 157361759 | 0.00010983 | 0.357043 | 0.26626 | 0.623302 |
| cg07602744 | PTPRN2 | Body;Body;Body | | 7 | 157484164 | 9.67E-05 | 0.378468 | 0.253576 | 0.632045 |
| cg19241327 | PTPRN2 | Body;Body;Body | | 7 | 157484559 | 0.00027859 | 0.317414 | 0.26168 | 0.579094 |
| cg15229124 | RBP1 | TSS1500;TSS1500;TSS1500 | 2 | 3 | 139258912 | 0.00015785 | 0.396838 | 0.391897 | 0.788735 |
| cg23363832 | RBP1 | TSS1500;TSS1500;TSS1500 | | 3 | 139258939 | 0.00040437 | 0.314784 | 0.264673 | 0.579457 |
| cg16425038 | REC8 | TSS200;TSS200 | 2 | 14 | 24641194 | 0.0001984 | 0.322219 | 0.298019 | 0.620239 |
| cg07516252 | REC8 | TSS200;TSS200 | | 14 | 24641201 | 0.00016328 | 0.318927 | 0.314202 | 0.633129 |
| cg24653181 | RHCG | Body | 2 | 15 | 90039582 | 0.00012771 | 0.323774 | 0.271058 | 0.594831 |
| cg18726691 | RHCG | 1stExon | | 15 | 90039613 | 9.79E-05 | 0.422424 | 0.312011 | 0.734435 |
| cg27507295 | RHCG | 1stExon;5'UTR | 6 | 15 | 90039794 | 9.70E-05 | 0.340755 | 0.318425 | 0.659179 |
| cg07485916 | RHCG | TSS200 | | 15 | 90039805 | 0.00011532 | 0.311905 | 0.31165 | 0.623554 |
| cg10721782 | RHCG | TSS200 | | 15 | 90039809 | 0.0001318 | 0.303272 | 0.315199 | 0.618471 |
| cg11332236 | RHCG | TSS200 | | 15 | 90039818 | 0.00011848 | 0.404421 | 0.255485 | 0.659906 |
| cg01837657 | RHCG | TSS200 | | 15 | 90039822 | 0.00014971 | 0.355455 | 0.269048 | 0.624503 |
| cg01524893 | RICH2 | Body | | 17 | 12877227 | 0.00014796 | 0.319803 | 0.191021 | 0.510824 |
| cg17278864 | RICH2 | Body | | 17 | 12877321 | 0.00014252 | 0.385465 | 0.251436 | 0.636901 |
| cg06766427 | RICH2 | Body | | 17 | 12877546 | 0.00012001 | 0.352222 | 0.266145 | 0.618367 |
| cg16585682 | SCGB3A1 | Body | 6 | 5 | 180017623 | 0.00020905 | 0.30669 | 0.211952 | 0.518642 |
| cg18652367 | SCGB3A1 | Body | | 5 | 180017689 | 9.49E-05 | 0.31488 | 0.177195 | 0.492075 |
| cg22859061 | SCGB3A1 | TSS200 | | 5 | 180018503 | 0.00022846 | 0.369193 | 0.417041 | 0.786233 |
| cg15457613 | SCGB3A1 | TSS200 | | 5 | 180018515 | 0.00015809 | 0.32099 | 0.240245 | 0.561235 |
| cg01934626 | SCGB3A1 | TSS200 | | 5 | 180018562 | 0.00012306 | 0.351134 | 0.261144 | 0.612278 |
| cg11916729 | SCGB3A1 | TSS200 | | 5 | 180018564 | 0.00016057 | 0.337932 | 0.24236 | 0.580292 |
| cg26331172 | SEMA6C | 5'UTR | 2 | 1 | 151118299 | 0.00015618 | 0.321636 | 0.373049 | 0.694685 |
| cg13331550 | SEMA6C | 5'UTR | | 1 | 151118416 | 0.00011532 | 0.354328 | 0.263412 | 0.61774 |

| | | | | | | | | | |
|---|---|---|---|---|---|---|---|---|---|
| cg25003777 | SERPINB1 | 5'UTR | 2 | 6 | 2841626 | 0.00012263 | 0.35513 | 0.211325 | 0.566455 |
| cg07635623 | SERPINB1 | 5'UTR | | 6 | 2841875 | 0.00016727 | 0.344736 | 0.232792 | 0.577528 |
| cg14610403 | SGIP1 | TSS1500 | 2 | 1 | 66998812 | 0.00015214 | 0.325295 | 0.282911 | 0.608206 |
| cg17692242 | SGIP1 | TSS1500;TSS1500 | | 1 | 66999038 | 0.00012913 | 0.316554 | 0.178749 | 0.495303 |
| cg18582992 | SHF | Body | 3 | 15 | 45479642 | 0.00023206 | 0.351804 | 0.330518 | 0.682323 |
| cg24033558 | SHF | Body | | 15 | 45479755 | 0.00015761 | 0.379363 | 0.242503 | 0.621865 |
| cg17102582 | SHF | 1stExon;1stExon;1stExon;Body | | 15 | 45479792 | 0.00016854 | 0.383399 | 0.28276 | 0.666159 |
| cg24302235 | SLC2A2 | TSS1500 | 4 | 3 | 170746073 | 0.00013983 | 0.370331 | 0.229622 | 0.599953 |
| cg01146875 | SLC2A2 | TSS1500 | | 3 | 170746172 | 0.00010983 | 0.404105 | 0.300081 | 0.704186 |
| cg07139301 | SLC2A2 | TSS1500 | | 3 | 170746207 | 0.00014339 | 0.402468 | 0.241118 | 0.643586 |
| cg14777768 | SLC2A2 | TSS1500 | | 3 | 170746265 | 0.00013333 | 0.385765 | 0.33779 | 0.723555 |
| cg12763828 | SLC2A5 | 5'UTR;;1stExon;1stExon;Body | 2 | 1 | 9129646 | 0.00011592 | 0.317679 | 0.307328 | 0.625007 |
| cg00310940 | SLC2A5 | 5'UTR;1stExon;1stExon;Body | | 1 | 9129648 | 0.0001183 | 0.311853 | 0.277534 | 0.589388 |
| cg11417025 | SOSTDC1 | TSS200 | 2 | 7 | 16505589 | 0.00013264 | 0.301518 | 0.271052 | 0.57257 |
| cg07220448 | SOSTDC1 | TSS200 | | 7 | 16505592 | 0.00012299 | 0.30828 | 0.321663 | 0.629943 |
| cg04265797 | SSTR1 | 3'UTR | 2 | 14 | 38679952 | 0.0001235 | 0.302102 | 0.254877 | 0.55698 |
| cg04573550 | SSTR1 | 3'UTR | | 14 | 38680313 | 0.00012233 | 0.323715 | 0.284751 | 0.608466 |
| cg01287975 | TAC1 | TSS200;TSS200;TSS200;TSS200 | 4 | 7 | 97361241 | 0.0003605 | 0.31368 | 0.393335 | 0.707015 |
| cg11873482 | TAC1 | TSS200;TSS200;TSS200;TSS200 | | 7 | 97361244 | 0.00035903 | 0.333668 | 0.309884 | 0.643551 |
| cg17437939 | TAC1 | TSS200;TSS200;TSS200;TSS200 | | 7 | 97361252 | 0.00033623 | 0.311081 | 0.433034 | 0.744114 |
| cg16288089 | TAC1 | 5'UTR;1stExon;1stExon;5'UTR; | | 7 | 97361408 | 0.00111929 | 0.302389 | 0.293885 | 0.596274 |
| cg24098326 | TACC2 | Body;TSS200;TSS200;Body | 2 | 10 | 123923050 | 0.00014922 | 0.344916 | 0.230564 | 0.575479 |
| cg06966660 | TACC2 | Body;TSS200;TSS200;Body | | 10 | 123923066 | 0.0002289 | 0.332565 | 0.266731 | 0.599296 |
| cg20591112 | TIAM1 | TSS1500 | 3 | 21 | 32932324 | 0.00027392 | 0.314562 | 0.206979 | 0.521541 |
| cg13748469 | TIAM1 | TSS1500 | | 21 | 32932341 | 0.0004461 | 0.332761 | 0.206115 | 0.538875 |
| cg17458922 | TIAM1 | TSS1500 | | 21 | 32932343 | 0.00017255 | 0.36564 | 0.252924 | 0.618564 |
| cg27521476 | TLX1 | Body | 2 | 10 | 102896376 | 0.00018715 | 0.313291 | 0.325731 | 0.639022 |
| cg01175020 | TLX1 | Body | | 10 | 102896475 | 0.00037114 | 0.317286 | 0.337978 | 0.655263 |
| cg19548479 | TMEM106A | TSS200 | 5 | 17 | 41363737 | 0.00010235 | 0.36738 | 0.213878 | 0.581258 |

| | | | | | | | | | |
|---|---|---|---|---|---|---|---|---|---|
| cg24940138 | TMEM106A | TSS200 | | 17 | 41363741 | 0.00018369 | 0.354264 | 0.225549 | 0.579813 |
| cg03049782 | TMEM106A | TSS200 | | 17 | 41363891 | 8.85E-05 | 0.381729 | 0.226908 | 0.608636 |
| cg24008544 | TMEM106A | 5'UTR;1stExon | | 17 | 41363899 | 0.0001019 | 0.404569 | 0.192904 | 0.597473 |
| cg18222083 | TMEM106A | 5'UTR | | 17 | 41364007 | 0.00021079 | 0.367403 | 0.281573 | 0.648975 |
| cg23831143 | TNFRSF10C | TSS200 | 2 | 8 | 22960427 | 0.00021563 | 0.335777 | 0.218447 | 0.554224 |
| cg27090216 | TNFRSF10C | 5'UTR;1stExon | | 8 | 22960461 | 0.0001183 | 0.372096 | 0.241496 | 0.613592 |
| cg08396863 | TUBA4B | TSS1500;Body | 3 | 2 | 220117599 | 0.00011298 | 0.300615 | 0.256085 | 0.5567 |
| cg08378442 | TUBA4B | TSS1500;Body | | 2 | 220117704 | 0.0001318 | 0.318976 | 0.1786 | 0.497576 |
| cg16725050 | TUBA4B | TSS200;Body | | 2 | 220117771 | 9.49E-05 | 0.383664 | 0.266517 | 0.650181 |
| cg04825044 | TXNRD1 | TSS200 | 3 | 12 | 104609402 | 0.00012306 | 0.331409 | 0.290154 | 0.621563 |
| cg14142965 | TXNRD1 | TSS200 | | 12 | 104609432 | 0.0002778 | 0.38441 | 0.197577 | 0.581987 |
| cg17395064 | TXNRD1 | TSS200 | | 12 | 104609476 | 0.00015922 | 0.345096 | 0.271955 | 0.617051 |
| cg22538054 | USP44 | 5'UTR;5'UTR | 3 | 12 | 95941988 | 9.67E-05 | 0.491277 | 0.317345 | 0.808622 |
| cg03918605 | USP44 | TSS200;5'UTR;5'UTR | | 12 | 95942081 | 0.00015523 | 0.392395 | 0.233286 | 0.625681 |
| cg00256076 | USP44 | TSS200;5'UTR;5'UTR | | 12 | 95942086 | 0.00018174 | 0.358259 | 0.220572 | 0.578831 |
| cg23740882 | WFDC2 | TSS200 | 2 | 20 | 44098387 | 0.00024369 | 0.319419 | 0.237742 | 0.557161 |
| cg00980978 | WFDC2 | Body | | 20 | 44098724 | 0.00020396 | 0.315448 | 0.223485 | 0.538933 |
| cg27049766 | ZNF154 | 5'UTR;1stExon | 5 | 19 | 58220516 | 0.00015428 | 0.347937 | 0.319461 | 0.667399 |
| cg03234186 | ZNF154 | TSS200 | | 19 | 58220657 | 0.00014674 | 0.313877 | 0.331218 | 0.645095 |
| cg08668790 | ZNF154 | TSS200 | | 19 | 58220662 | 0.0001609 | 0.327252 | 0.302201 | 0.629453 |
| cg12506930 | ZNF154 | TSS200 | | 19 | 58220718 | 0.0001183 | 0.323238 | 0.334235 | 0.657473 |
| cg26465391 | ZNF154 | TSS200 | | 19 | 58220773 | 0.0001183 | 0.318484 | 0.326323 | 0.644807 |
| cg10783469 | ZNF577 | TSS200;TSS200;TSS200 | 4 | 19 | 52391234 | 0.00064328 | 0.319455 | 0.280978 | 0.600433 |
| cg16731240 | ZNF577 | TSS200;TSS200;TSS200 | | 19 | 52391250 | 0.00026924 | 0.328658 | 0.302673 | 0.631332 |
| cg23010048 | ZNF577 | TSS200;TSS200;TSS200 | | 19 | 52391257 | 0.00038348 | 0.301876 | 0.340959 | 0.642835 |
| cg11269599 | ZNF577 | TSS200;TSS200;TSS200 | | 19 | 52391304 | 0.00054431 | 0.308428 | 0.285639 | 0.594067 |
| cg25666433 | ZSCAN12 | Body;5'UTR | 3 | 6 | 28367279 | 0.000159 | 0.334584 | 0.213363 | 0.547947 |
| cg02622316 | ZSCAN12 | Body;5'UTR | | 6 | 28367410 | 0.00012797 | 0.397417 | 0.261093 | 0.65851 |
| cg23164203 | ZSCAN12 | Body;5'UTR;1stExon | | 6 | 28367475 | 0.00018991 | 0.302519 | 0.192559 | 0.495077 |
